# FRAX597, a PAK1 inhibitor, synergistically reduces pancreatic cancer growth when combined with gemcitabine

**DOI:** 10.1186/s12885-016-2057-z

**Published:** 2016-01-16

**Authors:** Dannel Yeo, Hong He, Oneel Patel, Andrew M. Lowy, Graham S. Baldwin, Mehrdad Nikfarjam

**Affiliations:** Department of Surgery, Austin Health, University of Melbourne, Heidelberg, VIC Australia; Department of Surgery, Division of Surgical Oncology, University of California at San Diego, Moores Cancer, La Jolla, CA USA

**Keywords:** Pancreatic adenocarcinoma, PAK1, Gemcitabine, Proliferation, Orthotopic murine model

## Abstract

**Background:**

Pancreatic ductal adenocarcinoma remains one of the most lethal of all solid tumours. Treatment options are limited and gemcitabine-based chemotherapy remains the standard of care. Although growing evidence shows that p21-activated kinase 1 (PAK1) plays a crucial role in pancreatic cancer, its role has not been fully elucidated. This study aimed to characterise the expression and functional relevance of PAK1 in pancreatic cancer.

**Methods:**

PAK1 expression was measured in pancreatic cancer specimens by immunohistochemistry and in pancreatic cancer cell lines by western blotting. The effect of inhibition of PAK1 by either shRNA knock-down (KD), or by a selective inhibitor, FRAX597, alone or in combination with gemcitabine, on cell proliferation and migration/invasion was measured by thymidine uptake and Boyden chamber assays, respectively. The effect on tumour growth and survival was assessed in orthotopic murine models.

**Results:**

PAK1 was expressed in all human pancreatic cancer samples tested, an7d was upregulated in all pancreatic cancer cell lines tested. PAK1 KD inhibited pancreatic cancer cell growth and survival, and increased sensitivity to gemcitabine treatment. AKT activity and HIF1α expression were also inhibited. FRAX597 inhibited pancreatic cancer cell proliferation, survival, and migration/invasion. When combined with gemcitabine, FRAX597 synergistically inhibited pancreatic cancer proliferation in vitro and inhibited tumour growth in vivo.

**Conclusions:**

These results implicate PAK1 as a regulator of pancreatic cancer cell growth and survival. Combination of a PAK1 inhibitor such as FRAX597 with cytotoxic chemotherapy deserves further study as a novel therapeutic approach to pancreatic cancer treatment.

**Electronic supplementary material:**

The online version of this article (doi:10.1186/s12885-016-2057-z) contains supplementary material, which is available to authorized users.

## Background

Pancreatic ductal adenocarcinoma remains one of the most lethal of all solid tumours. It is the fourth leading cause of cancer-related mortality in Australia and the United States and is expected to become the second leading cause of cancer-related deaths by 2030, based on current management with no significant treatment improvements [1]. The 5-year survival rate is less than 5 % and has not improved over the past few decades [2]. Although combinational chemotherapies exist such as FOLFIRINOX and gemcitabine with nab-paclitaxel, as a single agent, gemcitabine remains the standard of care for the treatment of pancreatic cancer in most countries [3]. Gemcitabine targets rapidly replicating cells by inhibiting DNA synthesis, but intrinsic and acquired chemoresistance is common. The limited treatment regimens and predicted increase in cancer-related mortality highlight the urgent need for the development of effective therapies based on our understanding of the molecular mechanisms involved in pancreatic cancer.

The most frequent and earliest mutation in pancreatic cancer is the Kras mutation, present in over 95 % of pancreatic cancer patients [4]. This mutation results in a constitutively active p21 protein, Ras, which can activate a number of signalling pathways, including other p21 proteins such as Cdc42 and Rac, through direct and indirect mechanisms [5]. These p21 proteins can then activate the p21-activated kinases (PAKs). Although there is evidence for the activation of PAKs by Kras-driven pathways, other non-Kras-driven pathways or indirect Kras mechanisms may also activate PAKs.

PAKs are a family of non-receptor serine/threonine kinases, which mediate many effector functions from cell cycle and DNA transcription to cell adhesion and motility [6]. There are six isoforms of PAKs, divided into two groups: Group 1 contains PAK1, 2 and 3, and Group 2 contains PAK4, 5 and 6. Of the six isoforms, PAK1 is the best documented and has been found to be up-regulated in a number of cancers [7], including pancreatic cancer [8]. PAK1 is also up-regulated in pancreatic cancer cell lines when expression of MUC13, a transmembrane mucin, is increased [9]. We have previously found that a non-selective PAK inhibitor, glaucarubinone, reduced pancreatic cancer growth, and that treatment in combination with gemcitabine resulted in synergistic inhibition [10]. The role of PAK1 in pancreatic cancer and its therapeutic potential have not been fully elucidated.

FRAX597 is a small-molecule pyridopyrimidinone that targets group 1 PAKs through binding to the ATP-binding site [11]. Although it has been found to preferentially target group 1 PAKs, FRAX597 also inhibits other kinases such as RET, YES1, TEK, and CSF1R [12]. Of the group 1 PAKs, FRAX597 selectively inhibits PAK1 with a kinase IC_50_ of 8 nM, compared to 13 nM and 19 nM with PAK2 and PAK3, respectively. No inhibition of the group 2 PAKs was observed [11]. FRAX597 inhibits proliferation in neurofibromatosis type 2 (NF2)-associated schwannomas [12], but has not been tested previously in pancreatic cancer. This study aimed to elucidate the role of PAK1 in pancreatic cancer, by examining the effects of reduction of PAK1 expression by shRNA knock-down, or PAK1 activity with the selective inhibitor FRAX597, on the growth and migration/invasion of pancreatic cancer cell lines in vitro, and in orthotopic murine models in vivo, alone and in combination with gemcitabine.

## Methods

### Cells and reagents

The human PANC-1, MiaPaCa-2 and BxPC-3 pancreatic cancer cell lines (American Type Culture Collection, Manassas, VA) and the murine Pan02 (Division of Cancer Treatment and Diagnosis Tumor Repository, NCI, Frederick, MD), and LM-P (obtained from Andrew Lowy (Moores Cancer Center, University of California, San Diego, CA) [13]) pancreatic cancer cell lines were cultured in Dulbecco’s Modified Eagle’s Medium (DMEM) supplemented with 10 % FBS (fetal bovine serum: Hyclone Laboratories Inc., Scoresby, Australia). Normal immortalised human pancreatic duct epithelial (HPDE) cells (obtained from M.S Tsao (Ontario Cancer Institute, Ontario, Canada)) were cultured in Keratinocyte serum-free medium supplemented with bovine pituitary extract (BPE) and epidermal growth factor (EGF). All cells were cultured in a 37 °C incubator with 5 % CO_2_. Cells were tested regularly for mycoplasma contamination and were not passaged more than 30 times or for more than 6 months after resuscitation.

FRAX597 was purchased from SYNthesis (Parkville, Australia) and gemcitabine was purchased from Sigma-Aldrich (Castle Hill, Australia).

### Immunohistochemistry (IHC) on human pancreatic cancer samples

Human tissue collection was approved by the Austin Health Human Research Ethics Committee (H2013/04953) and informed consent was obtained from all participants. Samples of 10 human pancreatic cancers and adjacent normal pancreas were collected from patients undergoing pancreatic cancer resection at Austin Health, and confirmed to have pancreatic ductal adenocarcinoma by two independent pathologists. For PAK1 IHC, sample sections were incubated with 3 % hydrogen peroxide in methanol for 10 min at room temperature to quench endogenous peroxidase activity. Antigens were retrieved by incubation in 10 mM citrate buffer and blocked in 5 % horse serum. Sections were incubated with antibody against PAK1 (Santa Cruz Biotechnology, Dallas, TX) or IgG. Sections were visualised using an ENvision Plus polymer-based detection kit (Dako, Botany, Australia). The slides were then counter-stained and images were taken with a NIKON Coolscope (Coherent Scientific, Hilton, Australia).

### shRNA transfection

To obtain PAK1 knock-down (KD) clones, PANC-1 and MiaPaCa-2 cells were transfected with SureSilencing shRNA plasmids for human PAK1 (SABioscience, Doncaster, Australia), or with a scrambled sequence as a negative control (NC), using Lipofectamine2000 (Invitrogen, Mulgrave, Australia), according to the manufacturer's instructions. Stable clones were selected with geneticin (G418; 1 mg/ml). PAK1 protein expression was detected by western blot.

### Western blot

Proteins in cell lysates were detected with antibodies against phospho-PAK1 (Santa Cruz Biotechnology), PAK1, phospho-AKT, AKT, HIF1α (BD Biosciences, North Ryde, Australia), and GAPDH. Antibodies were from Cell Signalling Technology (Arundel, Australia), unless otherwise stated. Bound antibodies were visualized using ECL reagents (GE Healthcare, Amersham, UK), and the density of each band was analysed using Multigauge computer software (Berthold, Bundoora, Australia). HIF1α expression was determined in cells cultured under normoxia or hypoxia (1 % O_2_).

### Cell proliferation, cell survival and combination index

Cell proliferation and survival was measured using ^3^H-thymidine incorporation and withdrawal assays, respectively, as previously described [14]. Growth curves were fitted based on a log-scale using MATLAB (MathWorks, Natick, MA) and differences in proliferation were evaluated by comparison of growth rates (expressed as %/h). Assessment of proliferation with FRAX597 and the combination of FRAX597 with gemcitabine was measured as previously described [10]. For assessment of cell survival with FRAX597, cells were seeded with increasing concentrations of FRAX597 for 24 h without serum.

The combined effects of FRAX597 and gemcitabine were evaluated using the Chou-Talalay method [15] as previously described [10]. The CalcuSyn program (Biosoft, Cambridge, UK) was used to calculate the combination index (CI) for each drug affected fraction (Fa). The CI value is interpreted as: <1, synergistic; =1, additive; >1, antagonistic.

### Cell migration/invasion

Cell migration/invasion was measured using the Transwell Boyden chamber assay as previously described [14]. Cells were seeded into the upper chambers of the inserts (ThinCert™, 8 μm pore size; Greiner Bio-One, Frickenhausen, Germany) with increasing concentrations of FRAX597. After 24 h, membranes were fixed and stained with Quick-Dip (Fronine, Riverstone, Australia) and 24 fields were counted at 40 times magnification using a NIKON Coolscope.

### Murine orthotopic pancreatic cancer model

All mice experiments were approved by the Austin Health Animal Research Ethics Committee (A2013/04898). Pan02 cells were implanted orthotopically in the pancreatic head or tail as previously described [16]. For assessment of tumour growth, 28 mice were implanted with cells in the pancreatic tail and monitored for 30 days. 7 mice per treatment group were randomly allocated to the four treatment groups: control, intraperitoneal (i.p.) injection of saline every other day; FRAX597 alone, FRAX597 (3 mg/kg) i.p. every other day; gemcitabine alone, gemcitabine (40 mg/kg) i.p. twice weekly; and combination of FRAX597 and gemcitabine, following the individual treatments as described above. A single investigator measured the dimensions of all tumours, at the endpoint, using micro-calipers, in a double-blinded manner. Tumour volume was calculated using the formula for ellipsoid tumours: V = L x W x H x (π/6) where L was the longest distance from right to left; W, the largest dorsal/ventral diameter; and H, the largest rostral/caudal diameter. For assessment of survival, 54 mice were implanted with cells in the head of the pancreas. Mice were monitored based on health score for up to 45 days and euthanased when a poor health score was reached. Mice were treated with either control, gemcitabine alone, or combination of FRAX597 and gemcitabine, as described above. An initial study was undertaken with 24 mice, with 12 mice per group for control or gemcitabine treatment alone. A second study was undertaken with 30 mice, with 13 mice per group for gemcitabine alone or combination treatment, and the remaining 4 mice allocated to the control group. A collated Kaplan-Meier survival curve was plotted, and the two studies were analysed together using stratified Cox regression analysis (SPSS; IBM, New York, NY).

### Statistical analysis

All values are expressed as means ± standard error. Experiments were done in duplicate and data collated from three independent experiments. Results were analysed using student’s *t*-test or one-way ANOVA (SPSS). Differences between two means with p < 0.05 were considered significant.

## Results

### PAK1 is expressed in human pancreatic cancer and upregulated in pancreatic cancer cell lines

**Fig. 1:**
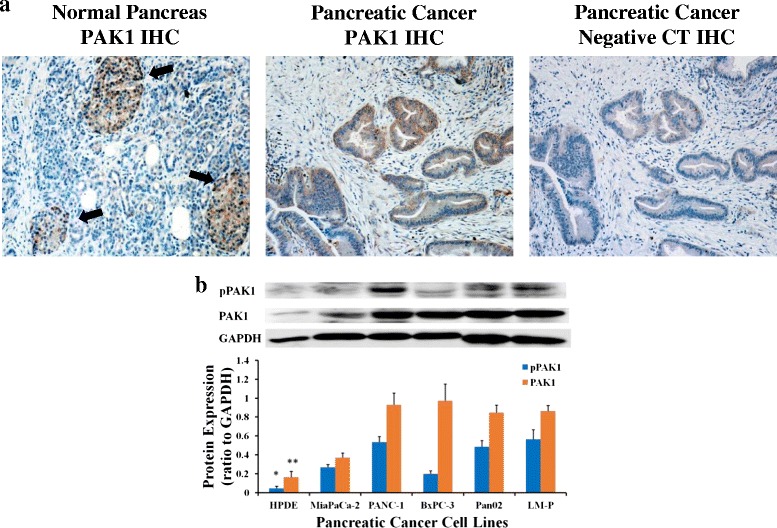
PAK1 is up-regulated in pancreatic cancer specimens and pancreatic cancer cell lines. (**a**) Acinar and ductal cells in normal pancreas are not stained for PAK1 by immunohistochemistry (IHC), but islet cells (arrow) are positive. Magnification: x200. Pancreatic ductal adenocarcinomas stain more strongly for PAK1 than the negative control (CT). Magnification: x100. (**b**) The normal pancreas cell line, HPDE, expressed low levels of phospho-PAK1 (active form) and PAK1 as detected by western blotting. All pancreatic cancer cell lines, MiaPaCa-2, PANC-1, BxPC-3, Pan02 and LM-P expressed phospho-PAK1 and PAK1. The data represent mean ± SEM, summarised from three independent experiments. * *p* < 0.05; ** p < 0.01, compared to all other pancreatic cancer cell lines

PAK1 staining of pancreatic ductal adenocarcinoma cells was observed in all 10 human pancreatic cancer samples tested (Fig. 1a). In corresponding normal pancreas samples, islet cells stained for PAK1, however, staining was absent in acinar and ductal epithelial cells. Expression of PAK1, and of the phosphorylated, active form of PAK1, was detected in low levels in the normal HPDE cell line and was significantly lower when compared to all the pancreatic cancer cell lines. All human and murine pancreatic cancer cell lines tested expressed phosphorylated and total PAK1 (Fig. 1b).

### Inhibition of PAK1 by shRNA knock-down decreases proliferation and survival of pancreatic cancer cells

**Fig. 2:**
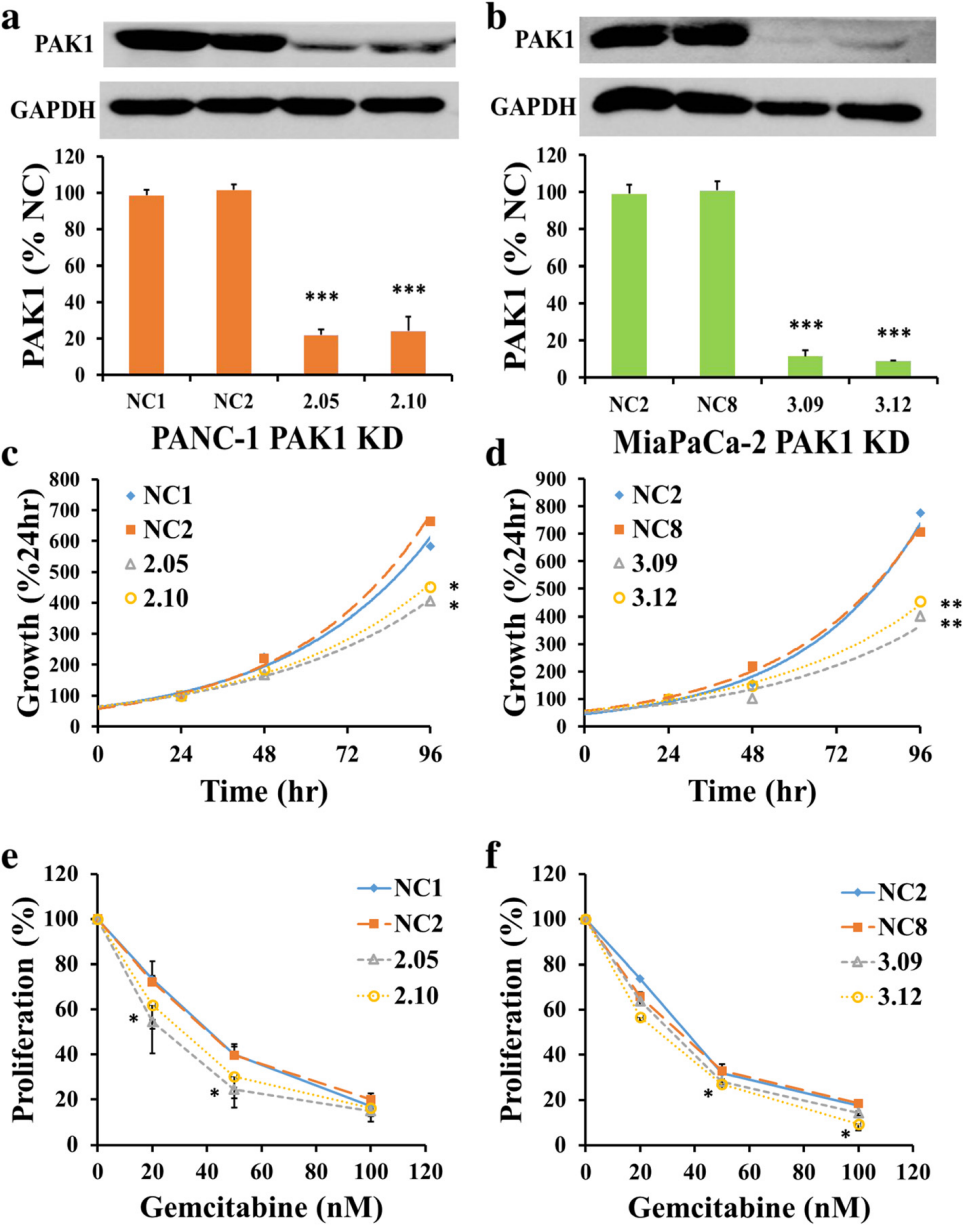
PAK1 knock-down (KD) inhibits proliferation and increases gemcitabine sensitivity. PANC-1 (**a**) and MiaPaCa-2 (**b**) PAK1 KD cells were generated using shRNA transfection. As detected by western blot, clones 2.05 and 2.10; and clones 3.09 and 3.12 for PANC-1 and MiaPaCa-2, respectively, expressed significantly less PAK1 than negative control (NC) clones, which had been transfected with a scrambled shRNA. The proliferation rate of the KD clones for both PANC-1 (**c**) and MiaPaCa-2 (**d**), measured by thymidine incorporation, was significantly lower after 96 h. Sensitivity of the KD clones to gemcitabine (20 nM and 50 nM for PANC-1 (**e**), and 50nM and 100nM for MiaPaCa-2 (**f**)) was significantly increased. The data represent mean ± SEM, summarised from three independent experiments. * *p* < 0.05; ** *p* < 0.01, *** *p* < 0.001, compared to either NC clone (only the higher p value of the two is presented)

The PAK1 protein concentrations in two PANC-1 PAK1 KD clones (2.05 and 2.10) were decreased to 22 % and 24 %, respectively, of the PAK1 protein concentrations of the corresponding NC cells, which had been transfected with scrambled sequences (Fig. 2a). Similarly, the PAK1 protein concentrations in two MiaPaCa-2 PAK1 KD clones (3.09 and 3.12) were decreased to 11 % and 9 %, respectively, of the PAK1 protein expression of the corresponding NC cells (Fig. 2b). The proliferation rate was significantly reduced in both PANC-1 (Fig. 2c) and MiaPaCa-2 (Fig. 2d) PAK1 KD cells compared to the corresponding NC cells. The growth rate of two clones of PANC-1 PAK1 KD cells (1.9 %/h and 2.1 %/h) was significantly less than two clones of NC cells (2.4 %/h and 2.6 %/h) (Table 1). A similar difference was observed in the MiaPaCa-2 PAK1 KD cells.

**Table 1:** PAK1 knock-down (KD) inhibits proliferation and increases gemcitabine sensitivity

	Growth rate (%/h)		Gemcitabine IC_50_ (nM)	
	PANC-1	MiaPaCa-2	PANC-1	MiaPaCa-2
NC1	2.4	2.9	26 ± 2	29 ± 1
NC2	2.6	2.7	39 ± 1	28 ± 1
KD1	1.9 *	2.0 **	20 ± 2 **	26 ± 1 *
KD2	2.1 *	2.1 **	21 ± 2 *	25 ± 2 *

NC1 and NC2 indicate PANC-1 NC clones NC1 and NC2; and MiaPaCa-2 NC clones NC2 and NC8 respectively. KD1 and KD2 indicate PANC-1 KD clones 2.05 and 2.10; and MiaPaCa-2 KD clones 3.09 and 3.12 respectively. * *p* < 0.05; ** *p* < 0.01, compared to either NC clone (only the higher p value of the two is presented)

### Inhibition of PAK1 by shRNA knock-down sensitises pancreatic cancer cells to gemcitabine

Proliferation of PANC-1 (Fig. 2e) and MiaPaCa-2 (Fig. 2f) PAK1 KD cells in the presence of gemcitabine at concentrations of 20 nM and 50 nM was inhibited to a greater extent than the corresponding NC cells. The IC_50_ values of two clones of PANC-1 PAK1 KD cells (20 nM and 21 nM) (Table 1), were significantly less than the values for NC cells (26 nM and 39 nM). Similarly, the IC_50_ values of two clones of MiaPaCa-2 PAK1 KD cells (26 nM and 25 nM) (Table 1) were significantly less than the values for NC cells (29 nM and 28 nM).

### Inhibition of PAK1 by shRNA knock-down reduces AKT activity and HIF-1α expression

**Fig. 3:**
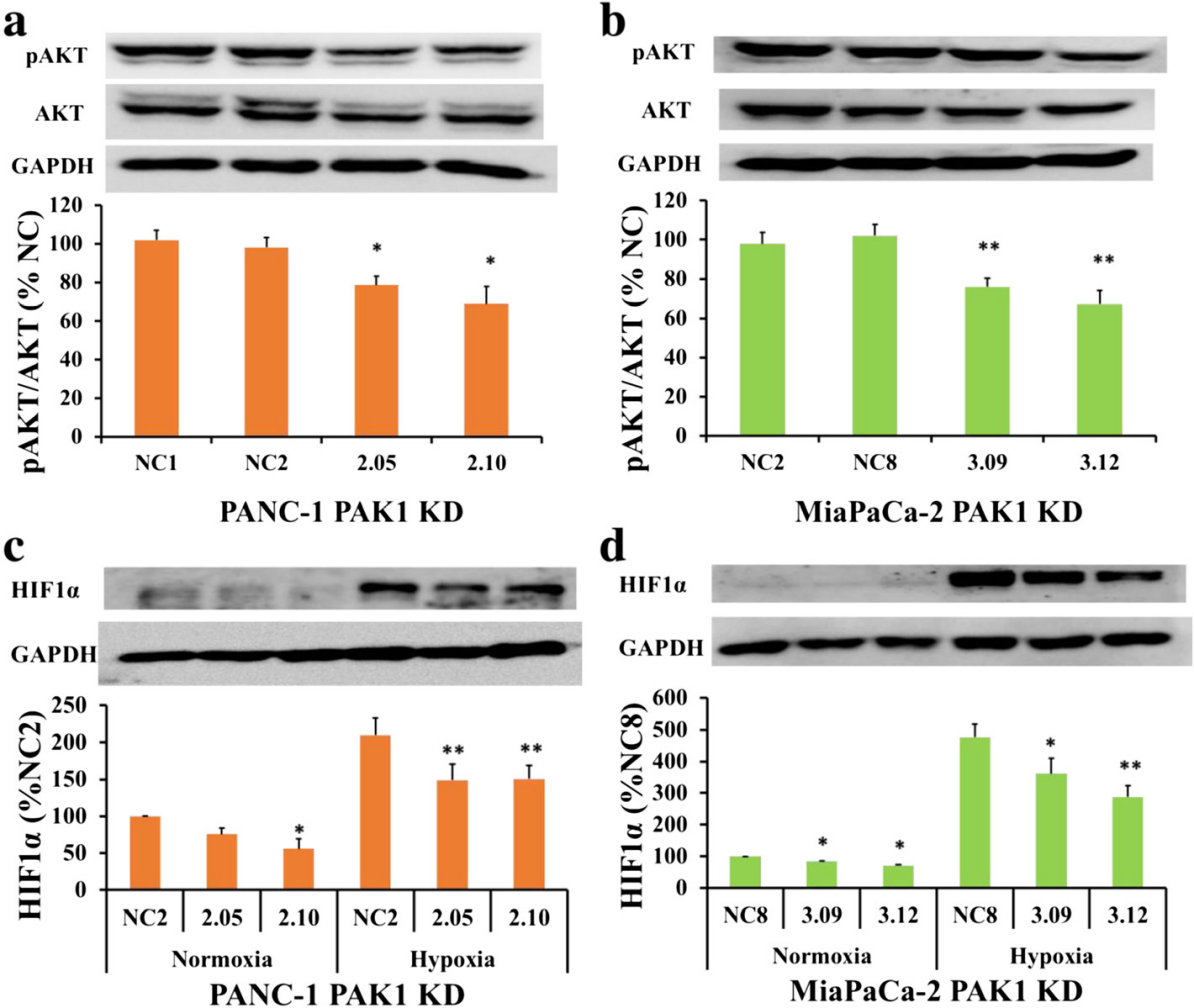
PAK1 knock-down (KD) inhibits expression of AKT and HIF1α. Expression of phospho-AKT (pAKT) was significantly reduced in the PAK1 KD clones: 2.05 and 2.10 (PANC-1 (**a**)); and 3.09 and 3.12 (MiaPaCa-2 (**b**)), compared to the negative controls (NC), as assessed by western blot. HIF1α expression was reduced in PANC-1 (**c**) and MiaPaCa-2 (**d**) PAK1 KD clones under normoxia and hypoxia (1 % O_2_) conditions. The data represent mean ± SEM, summarised from three independent experiments. * *p* < 0.05; ** *p* < 0.01, *** *p* < 0.001, compared to either NC clone (only the higher p value of the two is presented)

AKT activity was significantly reduced in two clones of PANC-1 PAK1 KD cells, by 22 % and 31 % (Fig. 3a), and in two clones of MiaPaCa-2 PAK1 KD cells by 24 % and 33 % (Fig. 3b). HIF1α expression was significantly reduced in two clones of both PANC-1 (Fig. 3c) and MiaPaCa-2 (Fig. 3d) PAK1 KD cells compared to the NC cells under either normoxia or hypoxia.

### FRAX597 decreases proliferation and migration/invasion in pancreatic cancer cell lines

**Fig. 4:**
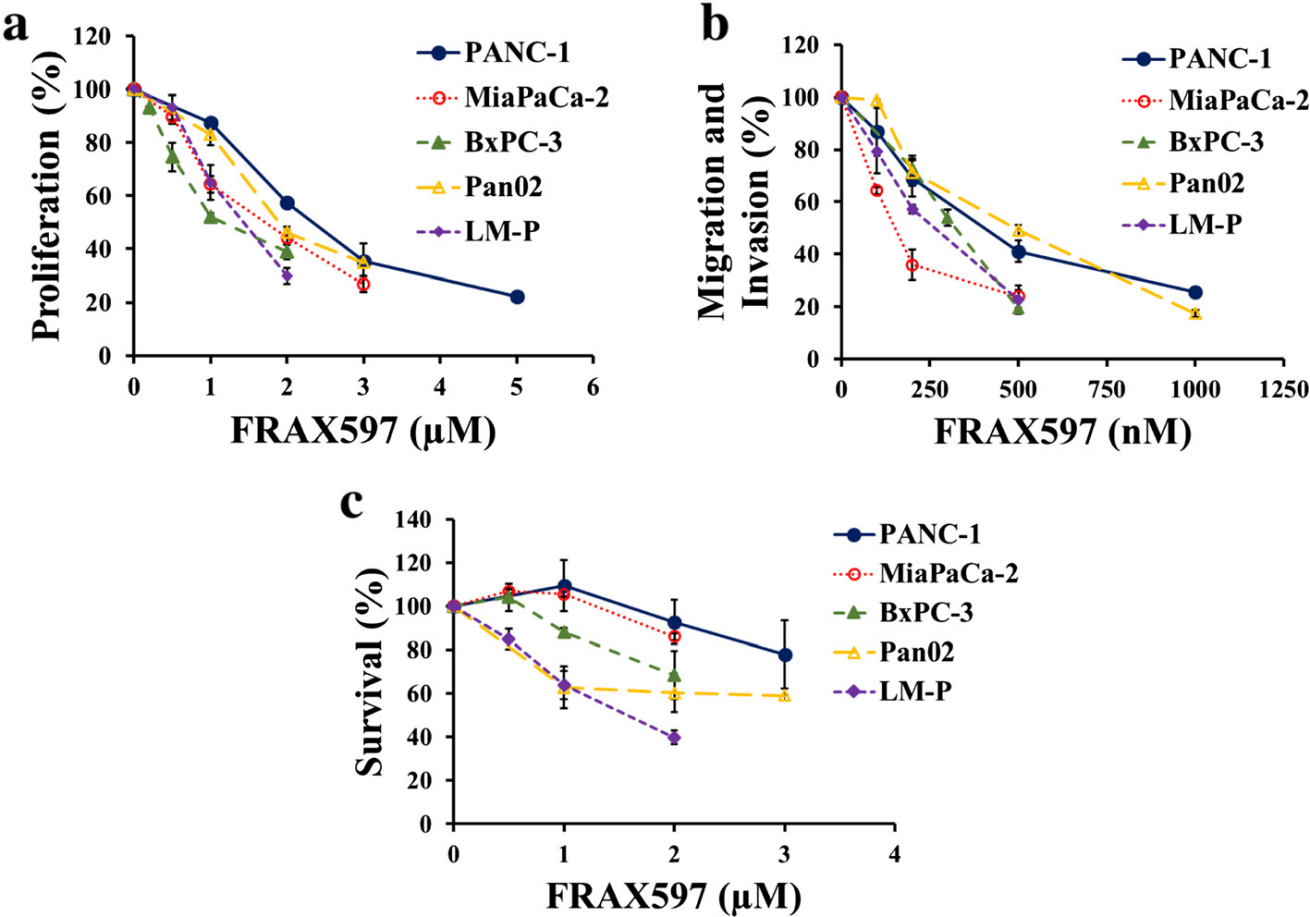
FRAX597 inhibits proliferation, migration/invasion, and survival. The effect of the selective group 1-PAK inhibitor FRAX597 on cell proliferation (**a**), cell migration/invasion (**b**) and cell survival (**c**) of the indicated human pancreatic cancer cell lines was measured using the thymidine-incorporation method, the Transwell Boyden chamber assay, and the thymidine-withdrawal method, respectively. The values for the untreated cells were taken as 100 %. The data represent mean ± SEM, summarised from three independent experiments. Significance is not shown for clarity

FRAX597 inhibited proliferation in all pancreatic cancer cell lines in a dose-dependent manner (Fig. 4a), with IC_50_ values between 650 nM for BxPC-3 cells and 2.0 μM for PANC-1 cells (Table 2). Similarly, FRAX597 inhibited migration and invasion in all pancreatic cancer cell lines in a dose-dependent manner (Fig. 4b), with IC_50_ values between 105 nM for MiaPaCa-2 cells and 605 nM for Pan02 cells (Table 2). FRAX597 inhibited survival of LM-P cells in the absence of FBS in a dose-dependent manner with an IC_50_ value of 1.10 μM (Fig. 4c). Significant inhibition of survival of PANC-1, MiaPaCa-2, BxPC-3, and Pan02 cells was only observed at concentrations greater than 1 μM.

**Table 2:** IC_50_ values for inhibition of proliferation and migration/invasion by FRAX597, and of proliferation by gemcitabine

	FRAX IC_50_ proliferation (μM)	FRAX IC_50_ migration/invasion (nM)	Gemcitabine IC_50_ proliferation (nM)
PANC-1	2.0 ± 0.2	290 ± 70	33 ± 3
MiaPaCa-2	1.4 ± 0.4	105 ± 10	30 ± 3
BxPC-3	0.65 ± 0.1	330 ± 45	5 ± 1
Pan02	1.4 ± 0.1	605 ± 80	80 ± 10
LM-P	1.1 ± 0.2	150 ± 25	16 ± 2

IC_50_ values were obtained by fitting the data in Fig. 3 and Additional file 2: Figure S2 using Sigmaplot

### FRAX597 synergises with gemcitabine in inhibiting pancreatic cancer cell growth

**Fig. 5:**
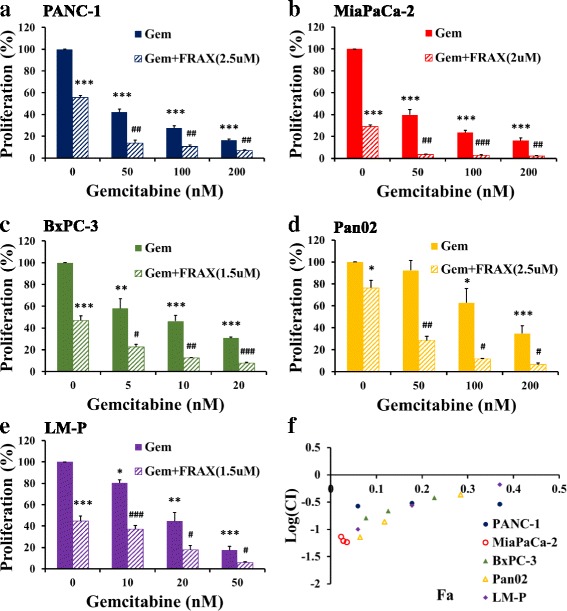
FRAX597 synergises with gemcitabine to inhibit proliferation. The effects of gemcitabine alone (Gem, solid bars), and gemcitabine after 20 h pre-treatment with FRAX597 (Gem + FRAX, striped bars), on proliferation of PANC-1 (**a**), MiaPaCa-2 (**b**), BxPC-3 (**c**), Pan02 (**d**), and LM-P (**e**) cells were assessed by thymidine incorporation. The concentration of FRAX597 used was based on the IC_50_ value determined in Fig. 4a. The combination index (CI), calculated by the Chou-Talalay method, was used to determine the mechanism of action of FRAX597 and gemcitabine (**f**). A value < 1 indicates synergistic inhibition. The data represent mean ± SEM, summarised from three independent experiments. * *p* < 0.05, ** *p* < 0.01, *** *p* < 0.001, compared to control or untreated cells. # *p* < 0.05, ## *p* < 0.01, ### *p* < 0.001 compared to the corresponding gemcitabine treatment

Gemcitabine alone inhibited proliferation in all pancreatic cancer cell lines (Fig. 5a-e) in a dose-dependent manner, with IC_50_ values between 5 nM for BxPC-3 cells and 80 nM for Pan02 cells (Table 2). A further reduction in proliferation was observed in all pancreatic cancer cell lines (Fig. 5a-e) when FRAX597 was combined with gemcitabine, compared to gemcitabine alone. The combination index, calculated for all pancreatic cancer cell lines (Fig. 5f), was less than 1, indicating that the effect was synergistic.

### Inhibition of pancreatic cancer cell growth by FRAX597 and gemcitabine is associated with reduced amounts of active PAK1

**Fig. 6:**
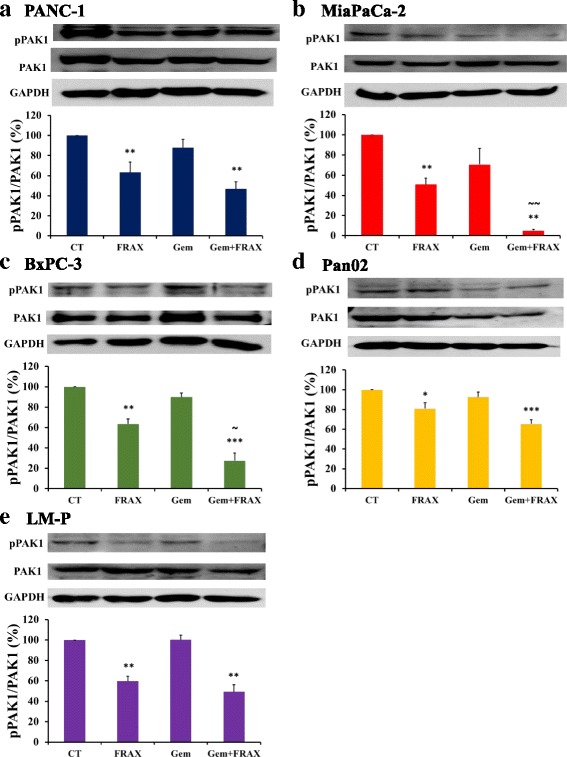
FRAX597 and gemcitabine reduce PAK1 activity. Expression of phospho-PAK1 (pPAK1) and PAK1 was measured in PANC-1 (**a**), MiaPaCa-2 (**b**), BxPC-3 (**c**), Pan02 (**d**), and LM-P (**e**) cells in the presence of FRAX597 (FRAX), gemcitabine (Gem), or the combination of FRAX597 and gemcitabine (Gem + FRAX) using western blot. Variations in protein loading were corrected by GAPDH expression, and the values for untreated control cells were taken as 100 %. The data represent mean ± SEM, summarised from three independent experiments. ** *p* < 0.01, *** *p* < 0.001, compared to control or untreated cells. ~ *p* < 0.05, ~ ~ *p* < 0.01 compared to FRAX597 treatment

The total amount of PAK1 and the amount of active phospho-PAK1 were measured using western blot after treatment with FRAX597 or gemcitabine, or the combination of FRAX597 and gemcitabine. The amount of active PAK1 was significantly reduced when treated with FRAX597 alone compared to control in all pancreatic cancer cell lines without affecting the amount of total PAK1 (Fig. 6a-e). No effect on PAK1 expression was observed after treatment with gemcitabine alone. For MiaPaCa-2 cells (Fig. 6b) and BxPC-3 cells (Fig. 6c), combined treatment with FRAX597 and gemcitabine resulted in significant further reduction of active PAK1 compared to the FRAX597 treatment alone. In contrast, in the other cell lines PANC-1 (Fig. 6a), Pan02 (Fig. 6d) and LM-P (Fig. 6e), no further reduction in active PAK1 expression was observed following the combination treatment.

### FRAX597 and gemcitabine inhibit pancreatic tumour growth in an orthotopic murine model

**Fig. 7:**
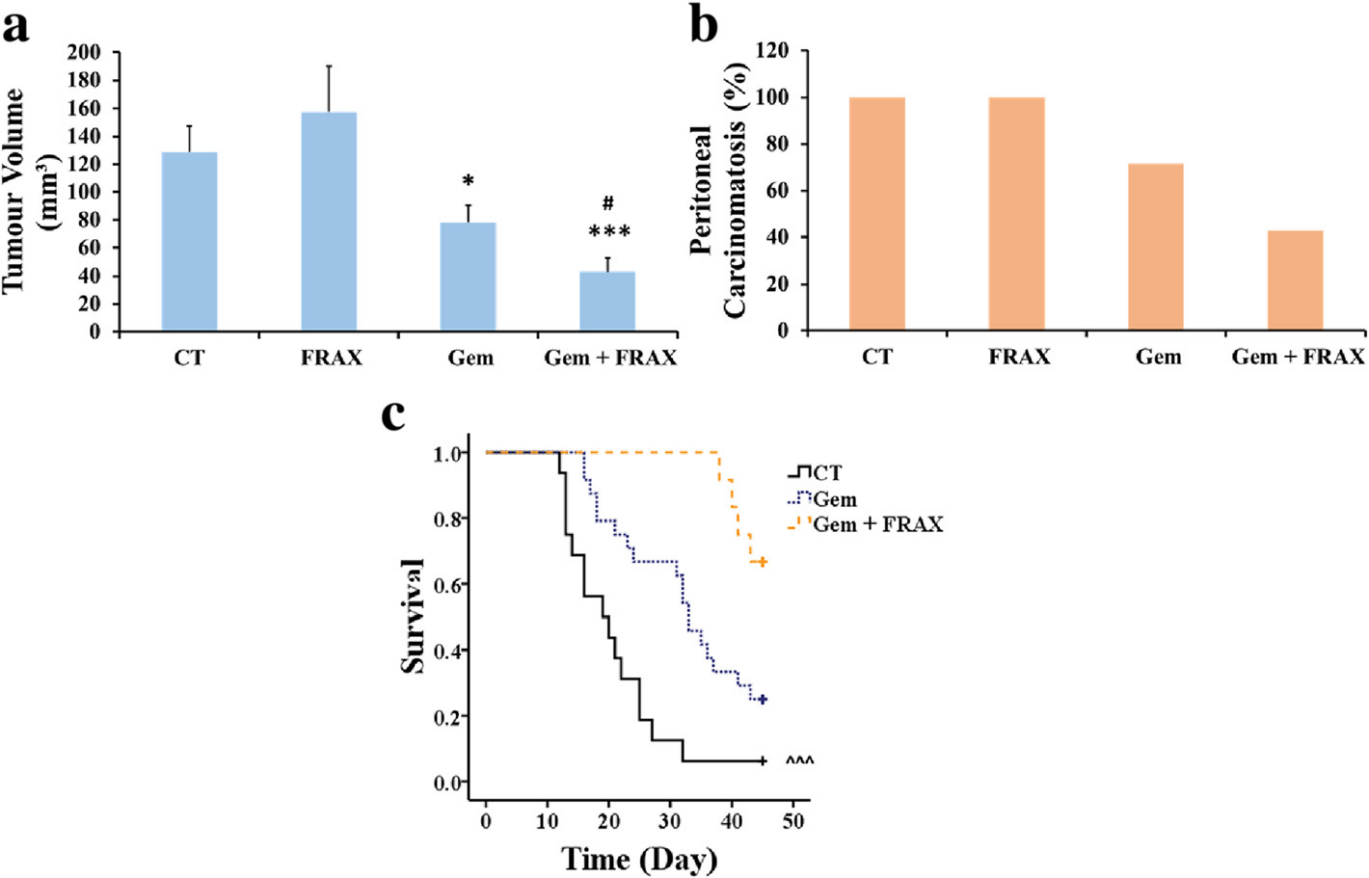
FRAX597 combined with gemcitabine inhibits tumour volume and increases survival *in vivo*. Pan02 murine pancreatic cancer cells were injected orthotopically into the tail (**a**-**b**) or head (**c**) of the pancreas of C57/Bl6 mice. Mice were treated with saline (control; CT), FRAX597 (FRAX), gemcitabine (Gem), or FRAX597 and gemcitabine (Gem + FRAX) at the doses given in the Materials and Methods section by intraperitoneal injection. Mice were euthanased after 30 days for the orthotopic pancreatic tail model and tumour volumes were measured (**a**), and scored for the presence of peritoneal carcinomatosis, or peritoneal spread (**b**). For assessment of survival, mice were euthanased after achieving a poor health score and the time to euthanasia plotted as a collated Kaplan-Meier curve (**c**). The data represent mean ± SEM. * *p* < 0.05, *** *p* < 0.001, compared to control. # *p* < 0.05 compared to gemcitabine treatment. ^^^ *p* < 0.001 compared to combination treatment (Gem + FRAX) using stratified Cox regression analysis

The tumour take rate was 100 % for both pancreatic head and pancreatic tail models. The survival rate following surgery was 100 % for the pancreatic tail model and over 95 % for the pancreatic head model. No difference in tumour volume was observed for mice treated with control or FRAX597 alone. Mice treated with gemcitabine alone had significantly reduced tumour volume when compared to control or FRAX597 alone, and a further significant reduction in tumour volume was observed for the mice treated with the combination of FRAX597 and gemcitabine (Fig. 7a). A similar trend was found when mice were evaluated for the presence of peritoneal carcinomatosis. 43 % of mice in the combined treatment group had peritoneal carcinomatosis compared to 71 % of mice in the gemcitabine treatment group, and 100 % of mice in the control and FRAX597 treatment groups (Fig. 7b).

Survival of mice in the combination treatment group was significant increased compared to the control group, as assessed by a stratified Cox regression analysis (Fig. 7c). A rates ratio of 7 was calculated, indicating that mice in the control group had a mortality rate 7 times greater than mice in the combination treatment group (Table 3). The rates ratio of 2.7 for mice in the gemcitabine alone group, compared to mice in the combination treatment group, was not statistically significant (*p* = 0.09).

**Table 3:** FRAX597 combined with gemcitabine increases survival in a mouse orthotopic pancreatic cancer model

Treatment	Rates ratio	95 % CI	*P* value
Control	7.0	1.8 ± 27.0	0.005
Gemcitabine	2.7	1.0 ± 8.7	0.09
Gemcitabine + FRAX	1.0 (ref)		

The overall statistics for the stratified Cox regression analysis were: *χ* ^2^ (2) = 9.9, *p* = 0.007

## Discussion

Our finding that PAK1 is expressed in pancreatic cancer is in agreement with previous studies [8, 17]. We confirmed that PAK1 was not expressed in normal pancreatic acinar or ductal cells, which are the likely progenitors of pancreatic cancer [18]. In contrast, PAK1 was expressed in the tumour tissue, in which the pancreatic ductal adenocarcinomas cells stained positive (Fig. 1a). All pancreatic cancer cell lines showed upregulation of PAK1 compared to the normal pancreas cell line, HPDE, regardless of Kras mutational status. The observation from BxPC-3 and Pan02, that are Kras wildtype cell lines, indicates that non-Kras mechanisms may result in the activation of PAK1. Further investigation will be required to elucidate those mechanisms [19, 20]. The emergence of PAK1 expression implies that PAK1 is involved in pancreatic carcinogenesis, however, its role and therapeutic potential have not been fully elucidated.

Reduction of PAK1 expression by shRNA knock-down (Fig. 2a-b) inhibited proliferation of the PANC-1 and MiaPaCa-2 pancreatic cancer cell lines (Fig. 2c-d), likely through modulation of the AKT pathway. These two human cell lines were chosen based on the PAK1 activity where PANC-1 is considered ‘high’ activity whilst MiaPaCa-2 is considered ‘low’ activity (Fig. 1b). This difference may contribute to the contrasting results in cell survival where a reduction was observed in PANC-1 PAK1 KD cells (Additional file 1: Figure S1A) but not in MiaPaCa-2 PAK1 KD cells (Additional file 1: Figure S1B). The suggestion that ‘high’ PAK1 expressing cells may be driving cell survival whereas ‘low’ PAK1 expressing cells may rely on other mechanisms to drive cell survival requires further investigation. The reduction in cell growth in PAK1 KD cells was associated with a decrease in AKT activity (Fig. 3a-b), but not in ERK activity (Additional file 1: Figure S1C-D). Our group has previously found that PAK1 mediated growth of colorectal cancer cell lines by both ERK and AKT pathways [14], while another group has found that PAK1 signalled preferentially through the ERK pathway to control skin cancer growth [11]. Thus, PAK1 signalling through AKT and ERK pathways is dependent on the cancer type, and our study suggests that PAK1 mediates pancreatic cancer cell growth through the AKT pathway rather than the ERK pathway.

PAK1 may play a role in the resistance of pancreatic cancer to hypoxia through regulation of HIF1α. The transcription factor HIF1α regulates oxygen delivery and metabolic adaptation to hypoxia and has been found to be a prognostic marker for pancreatic cancer [21]. Pancreatic tumours are known to be highly hypoxic, as they feature a dense desmoplastic reaction (stroma), which may contribute to pancreatic cancer invasion, metastasis, and resistance to therapy [22]. Thus, mediators of survival in response to a hypoxic challenge are attractive therapeutic targets for pancreatic cancer. Although, as far as we are aware, this is the first study to examine HIF1α as a downstream effector of PAK1 in pancreatic cancer, PAK1 has previously been linked to HIF1α in colorectal cancer [23]. The ability of PAK1 to contribute to pancreatic carcinogenesis via multiple signalling pathways enhances its potential as a therapeutic target.

PAK1 knock-down also enhanced the sensitivity of PANC-1 and MiaPaCa-2 cells to gemcitabine (Fig. 2e-f), as revealed by comparison of the IC_50_ values for inhibition of proliferation between control and knock-down clones (Table 1). Although gemcitabine remains a standard monotherapy treatment for pancreatic cancer patients, combining treatments with gemcitabine with the goal of decreasing chemotherapy-associated cytotoxicity and chemo-resistance and increasing survival has had varied results [3]. Previous studies have found that the PAK1 downstream effectors AKT and HIF1α could play a role in gemcitabine resistance through NFκB which limits gemcitabine uptake by decreasing nucleoside transporters such as hENT and hCNT [3]. Furthermore, PAK1 has been shown to regulate NFκB transcription upstream of fibronectin regulation in pancreatic cancer [8]. Although further investigation is required, the data presented herein supports the use of a PAK1 inhibitor combined with gemcitabine to limit gemcitabine cytotoxicity and chemo-resistance.

The group 1 PAK-selective inhibitor FRAX597 inhibited proliferation, migration/invasion, and survival of all pancreatic cancer cell lines tested (Fig. 4a-c). Although FRAX597 also inhibits other kinases such as RET, YES1, TEK, and CSF1R [12], the similar results obtained in the PAK1 knock-down experiments suggest that in this case PAK1 is indeed the relevant target. Furthermore, the IC_50_ values for proliferation are similar to the value observed in NF2-null Schwann cells [12]. However, the IC_50_ values for either proliferation or migration/invasion did not significantly correlate with the amount of active PAK1 in the pancreatic cancer cells (data not shown). This observation suggests that there may be a barrier (e.g. uptake at the cell membrane) that prevents realisation of the full potential for inhibition in intact cells. The presence of such a barrier could have contributed to the failure to detect any difference in tumour volume between the FRAX597-treated mice and the control mice in our *in vivo* study. Furthermore, the dense desmoplastic reaction may have also prevented the drug’s uptake by the tumour. These observations illustrate the importance of the microenvironment in assessment of a drug’s efficacy, as the *in vitro* cell culture conditions may not fully mimic the clinical setting.

Combination of the PAK1 inhibitor, FRAX597, with gemcitabine resulted in increased inhibition of PAK1 activity in some, but not all, of the pancreatic cancer cell lines tested (Fig. 6a-e). In all the pancreatic cancer cell lines tested, PAK1 activity was significantly decreased after treatment with FRAX597 alone, but no change in activity was observed after treatment with gemcitabine alone. Thus, combined treatment with FRAX597 and gemcitabine might be expected to inhibit PAK1 to the same extent as FRAX597 treatment alone, as was observed for PANC-1, Pan02 and LM-P cells. The significantly greater inhibition observed in MiaPaCa-2 and BxPC-3 cells after combination treatment provided clear evidence for synergy, although the mechanism for this is unclear. Interestingly, these two pancreatic cancer cell lines had the lowest phospho-PAK1 expression of all the pancreatic cancer cell lines tested. This observation suggests that phospho-PAK1 may be a predictive marker for gemcitabine response, as has recently been shown for PAK4 in pancreatic cancer [24].

Treatment with FRAX597 combined with gemcitabine significantly decreased tumour volume *in vivo* (Fig. 7a) and revealed a promising trend towards decreasing metastasis (Fig. 7b) and increasing survival (Fig. 7c). Furthermore, Ki67 staining of the tumours indicated that the difference in tumour volume was due to inhibition of proliferation (Additional file 2: Figure S2). Although liver metastasis is often observed in the orthotopic pancreatic tail murine model, a total of only three mice, from control and FRAX treatment groups, had liver metastases at sacrifice, so no comparison could be undertaken [16]. However, peritoneal carcinomatosis, or peritoneal spread, was present and was compared. As a difference in tumour volume was observed between animals treated with gemcitabine alone or with the combination of FRAX597 and gemcitabine, a decrease in peritoneal carcinomatosis and an increase in survival was expected, but significance was not reached. This may be due to the fact that the study was stopped early, before all mice were euthanised because of tumour-related illness. Although the potential clinical value of FRAX597 and the likely therapeutic benefit of targeting PAK1 are clearly established by the data in Fig. 4, longer studies are needed for a complete picture of the possible survival benefits of combination treatment.

## Conclusion

PAK1 is upregulated in human pancreatic cancer. Knock-down experiments indicated that PAK1 is required for proliferation and survival of human pancreatic cancer cell lines through AKT- and/or HIF1α-dependent pathway(s). Furthermore, PAK1 knock-down sensitised pancreatic cancer cells to gemcitabine. A group 1 PAK-specific inhibitor, FRAX597, inhibited proliferation, migration/invasion, and survival of human pancreatic cancer cell lines. When combined with gemcitabine, FRAX597 synergistically inhibited pancreatic cancer growth *in vitro* and *in vivo*. This study suggests the promise of inhibiting PAK1 function and defines areas for further investigation to clarify its potential value as a target for pancreatic cancer therapy.

### Ethics approval and consent to participate and publication

Human ethics approval was obtained from the Austin Health Human Research Ethics Committee (H2013/04953) and all participants gave consent to participate and for publication. All mice experiments were approved by the Austin Health Animal Research Ethics Committee (A2013/04898).

## Additional files

Additional file 1: Figure S1. PAK1 Knock-down (KD) effects on survival and ERK expression. PAK1 KD cells were measured in the presence (darker bars) and absence (lighter bars) of FBS to measure survival, using thymidine-withdrawal. Survival in PANC-1 PAK1 KD clones (A) was significantly lower but no difference was observed in MiaPaCa-2 PAK1 KD clones (B). No reduction in the expression of either phospho-ERK (pERK1/2) or total ERK (ERK1/2) was detected in either PANC-1 (C) or MiaPaCa-2 (D) PAK1 KD cells, as assessed by western blot. The data represent mean ± SEM, summarised from three independent experiments. *** *p* < 0.001, compared to the corresponding clone with FBS. (PPTX 433 kb) Additional file 2: Figure S2. FRAX597 and gemcitabine decreased Ki67 staining on orthotopic pancreatic tail tumours. Pan02 murine pancreatic tumours from the orthotopic pancreatic tail tumour model treated with saline (control; CT), FRAX597 (FRAX), gemcitabine (Gem), or FRAX597 and gemcitabine (Gem + FRAX) at the doses given in the Materials and Methods section, were fixed and stained for the proliferative marker, Ki67. Three representative images were taken from each treatment group. (PPTX 2275 kb)

## Abbreviations

BPE: bovine pituitary extract
CI: combination index
EGF: epidermal growth factor
Fa: affected fraction
HPDE: human pancreatic duct epithelial cells
IHC: immunohistochemistry
i.p.: intraperitoneal
KD: knock-down
NC: negative control
NF2: neurofibromatosis type 2
PAK1: p21-activated kinase 1
shRNA: short hairpin RNA
