# Supplementary figures and images for: FRAX597, a PAK1 inhibitor, synergistically reduces pancreatic cancer growth when combined with gemcitabine
Source: BMC Cancer. 2016 Jan 16;16:24. doi: 10.1186/s12885-016-2057-z (PMC4715347; doi:10.1186/s12885-016-2057-z)

## Slide 1
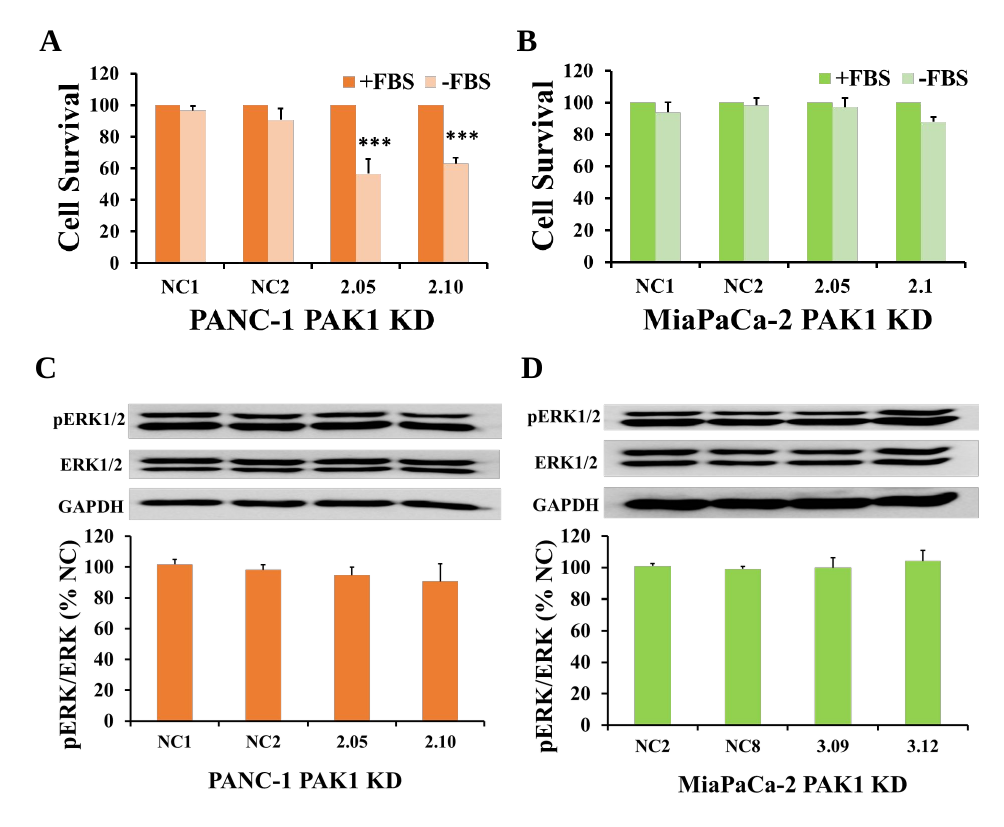

A
B
C
D

Supplement: Additional file 1: Figure S1. — PAK1 Knock-down (KD) effects on survival and ERK expression. PAK1 KD cells were measured in the presence (darker bars) and absence (lighter bars) of FBS to measure survival, using thymidine-withdrawal. Survival in PANC-1 PAK1 KD clones (A) was significantly lower but no difference was observed in MiaPaCa-2 PAK1 KD clones (B). No reduction in the expression of either phospho-ERK (pERK1/2) or total ERK (ERK1/2) was detected in either PANC-1 (C) or MiaPaCa-2 (D) PAK1 KD cells, as assessed by western blot. The data represent mean ± SEM, summarised from three independent experiments. *** p < 0.001, compared to the corresponding clone with FBS. (PPTX 433 kb) [file 12885_2016_2057_MOESM1_ESM.pptx]

## Slide 1
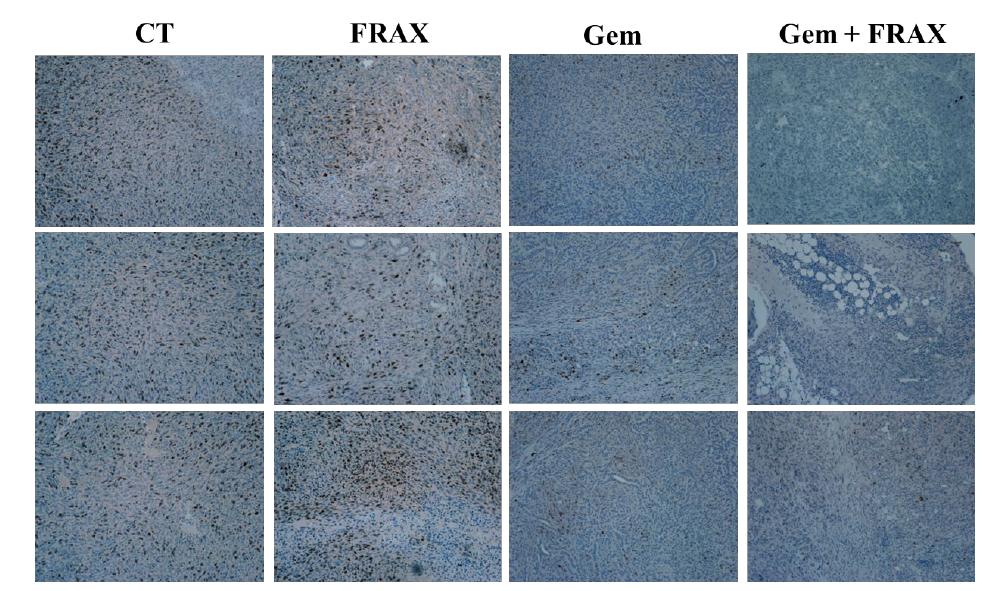

Supplement: Additional file 2: Figure S2. — FRAX597 and gemcitabine decreased Ki67 staining on orthotopic pancreatic tail tumours. Pan02 murine pancreatic tumours from the orthotopic pancreatic tail tumour model treated with saline (control; CT), FRAX597 (FRAX), gemcitabine (Gem), or FRAX597 and gemcitabine (Gem + FRAX) at the doses given in the Materials and Methods section, were fixed and stained for the proliferative marker, Ki67. Three representative images were taken from each treatment group. (PPTX 2275 kb) [file 12885_2016_2057_MOESM2_ESM.pptx]
